# Value of exercise stress electrocardiography for stratification of exercise capacity and left ventricular systolic and diastolic function on coronary slow flow: case-control study

**DOI:** 10.1186/s12872-019-01291-5

**Published:** 2019-12-12

**Authors:** Yonghuai Wang, Jinyang Li, Shuang Liu, Lixin Mu, Guangyuan Li, Hang Yu, Jun Yang, Chunyan Ma

**Affiliations:** 1 Department of Cardiovascular Ultrasound, First Hospital of China Medical University, No. 155 Nanjingbei Street, Shenyang, 110001 Liaoning China; 2 Department of Cardiology, First Hospital of China Medical University, Shenyang, Liaoning China

**Keywords:** Coronary slow flow phenomenon, Exercise stress electrocardiography, Left ventricular function, Echocardiography

## Abstract

**Background:**

Coronary slow flow phenomenon (CSFP) is an angiographic entity characterized by delayed coronary opacification in absence of evident obstructive lesion in the epicardial coronary artery. However, whether patients with CSFP exhibit differing responses to exercise is still not known. This study aimed to evaluate results of exercise stress electrocardiography (ExECG) and left ventricular (LV) function during exercise, and study the value of ExECG for stratification of exercise capacity and LV function in patients with CSFP.

**Methods:**

Thirty patients with CSFP and 24 controls were enrolled in the study. Diagnosis of CSFP was made by Thrombolysis in Myocardial Infarction frame count. ExECG and LV function measured by echocardiography at rest, during exercise and recovery phase were evaluated.

**Results:**

Negative ExECG was found in 24 (80%) patients with CSFP. At rest, LV global longitudinal strain (GLS) decreased and mitral average E/e’ increased in patients with CSFP compared with controls; however, there were no differences in these parameters between CSFP patients with negative ExECG and patients with positive ExECG. During exercise, CSFP patients with negative ExECG and controls had significantly increased LV GLS and decreased mitral average E/e’, but CSFP patients with positive ExECG had significantly decreased LV GLS and increased mitral average E/e’.

**Conclusions:**

About 80% patients with CSFP exhibited negative ExECG. CSFP patients with negative ExECG exhibited improved LV function but CSFP patients with positive ExECG exhibited impaired LV function during exercise. ExECG may aid in the stratification of exercise capacity and LV function in patients with CSFP.

## Background

Coronary slow flow phenomenon (CSFP) is an angiographic entity characterized by delayed coronary opacification in the absence of an evident obstructive lesion in the epicardial coronary artery [1]. CSFP is not an infrequent finding, with a reported incidence of approximately 1 to 7% in patients in whom coronary angiography is performed because of suspicious cardiovascular disease [2–4]. Rather than representing a simple angiographic observation, CSFP has been known to be associated with acute myocardial infraction, malignant ventricular fibrillation, or even aborted sudden death [5–7]. Moreover, our previous study revealed that left ventricular (LV) systolic and diastolic functions in patients with CSFP are impaired at rest [8, 9]. Therefore, patients with CSFP should receive close attention.

CSFP patients may experience recurrent angina, necessitating readmission to the coronary care unit or repeat coronary angiography for an acute exacerbation. However, it has been reported that patients with CSFP may have different kinds of angina [10]. Most patients with CSFP present with angina at rest, but other patients may present with exercise angina or mixed angina [10]. Therefore, we hypothesized that patients with CSFP may have differing results with exercise stress electrocardiography (ExECG). Moreover, whether patients with CSFP exhibit differing responses to exercise in LV function is still not precisely known.

It has been shown that ExECG can privode important value for risk stratification in suspected or known coronary heart disease who can exercise [11–13]. However, whether ExECG can aid in the stratification of exercise capacity and the LV function during exercise is not well understood. Distinguishing patients with CSFP with differing responses to exercise may provide the basis for differential management of these patients.

In view of the foregoing, we aimed to evaluate results of ExECG and LV function during exercise by echocardiography, and investigate the value of ExECG in the stratification of exercise capacity and LV function in patients with CSFP in the present study.

## Methods

### Study population

This was a case-control study performed in the cardiology department of our hospital between December 2017 and November 2018. Subjects were consecutively included in the study and had normal or near-normal (< 40% stenosis) coronary arteries on coronary angiography, which was performed because of angina, coronary risk factors or abnormal electrocardiography changes. The CSFP group consisted of individuals with a corrected thrombolysis in myocardial infarction (TIMI) frame count (TFC) exceeding 27 in one or more vessels [14]. The control group consisted of individuals with a corrected TFC not more than 27 in all vessels.

Patients having the following features were excluded: incalculable TFC; coronary artery spasm or ectasia; LV ejection fraction < 52% in males or < 54% in females [15]; any arrhythmia (atrioventricular conduction abnormalities, bundle branch block, ventricular preexcitation, atrial fibrillation, or paced rhythm); abnormal heart structure (congenital heart disease, cardiomyopathies, or valvular dysfunction); pericardial disease (pericardial effusion or constrictive pericarditis); previous history of myocardial infarction; uncontrolled hypertension (systolic blood pressure > 160 mmHg or diastolic blood pressure > 105 mmHg); hyperthyroidism; hypothyroidism; malignancy; autoimmune disease; infection; pulmonary, hepatic, and renal disorders; hematological disorders (anemia, bone marrow involved by neoplastic disease, or red blood cell transfusions); and a recent major operation (within 90 days).

All examinations were performed by investigators who had no information about the clinical status of the participants. All concomitant medications were stopped ≥12 h prior to the procedure. Written informed consent was obtained from all participants, and the study protocol was approved by China Medical University Ethics Committee and complied with the ethical guidelines of the 1975 Declaration of Helsinki.

### Coronary angiography and TFC calculation

Coronary angiography was performed using the General Electric Innova 3100 (Milwaukee, WI, USA) by the femoral approach in multiple angulated views. A standard Judkins technique was used in all the studied individuals with 5F Judkins catheters, and iohexol (350/100 mL) was used as a contrast agent and manually injected intravenously at the same rate of 3–4 mL/s for the left coronary artery and 2–3 mL/s for the RCA. TFC was used to quantitatively evaluate flow rates of each major coronary artery, including the left anterior descending artery (LAD), the left circumflex coronary artery (LCX), and the right coronary artery (RCA), according to the method first described by Gibson et al. [14]. TFC, recorded at 30 frames per second, was the number of frames from the second the contrast medium first appeared in the ostium of the coronary artery to the second it reached a distal coronary landmark. Because the LAD is usually longer than the LCX and RCA, the TFC of LAD is divided by 1.7 to obtain the corrected TFC of LAD (cLAD). The mean TFC for each subject was the average of TFC of RCA, LCX, and cLAD. The TFC was undertaken by two separate cardiologists and a third observer resolved any disagreement.

### Seattle angina questionnaire

Seattle Angina Questionnaire (SAQ) was collected at the time of study enrollment under the supervision of a trained cardiologist to assess symptoms of angina and their impact on quality of life. SAQ is a validated 19-item questionnaire that measures five key domains related to coronary artery disease: physical limitations, angina stability, angina frequency, treatment satisfaction, and quality of life. Scores range from 0 through 100 for all domains. Higher scores indicate fewer physical limitations due to angina, less angina, and better quality of life [16, 17].

### Exercise stress electrocardiography

Exercise testing was performed within 72 h after coronary angiography using standard Bruce protocol according to standard clinical practice. Heart rate and blood pressure were measured, and a 12-lead ECG was taken at rest, at each stage of the exercise protocol, and during recovery (≥6 min after exercise). Patients were motivated and encouraged to reach 85% of maximal predicted heart rate, until they reached an endpoint. Exercise endpoints included physical exhaustion, severe ischemia (severe chest pain, > 2 mm horizontal or downsloping ST depression), severe hypertension (systolic blood pressure > 240 mmHg or diastolic blood pressure > 110 mmHg), severe hypotension (decrease > 20 mmHg in systolic blood pressure from baseline), significant arrhythmia, or pre-syncope. Rate-pressure product and metabolic equivalents (METs) were recorded. Positive exercise stress ECG was defined as significant chest pain, hypotension, or ≥ 1 mm planar or downsloping ST depression in two or more leads of the same territory, during exercise or recovery. The results of ExECG were interpreted by two separate experienced cardiologists and a third observer resolved any disagreement.

### Resting and exercise stress echocardiography

According to the recommendations of the American Society of Echocardiography [15], standard echocardiographic examination was performed in the lateral decubitus position using a Vivid E9 ultrasound system (GE Healthcare, Waukesha, WI, USA) equipped with M5S phased-array probe. The two-dimensional cine loops were recorded for offline analysis using an EchoPAC work station (GE Healthcare).

All patients underwent a comprehensive echocardiography at rest. LV ejection fraction was measured by biplane Simpson method. In order to assess LV diastolic function, we measured left atrial (LA) volume index, mitral E, mitral A, mitral septal e’, mitral lateral e’, and tricuspid regurgitation velocity, and calculated mitral E/A, mitral average E’, and mitral average E/e’ [18].

Exercise stress echocardiography images were acquired immediately (within 90 s) after peak exercise from the second the patients lay in the bed and during recovery (≥6 min after exercise). The images included two-dimensional images from parasternal long-axis and three apical views (long-axis, four-chambers, and two-chambers), mitral valve flow Doppler spectrum, and mitral annular tissue Doppler spectrum. Immediate post-exercise two-dimensional images were obtained using a continuous imaging capture system and the images with best quality were chosen for analysis. Patients with poor imaging quality due to significant respiratory movements immediately after exercise were excluded. LV ejection fraction, LA volume index, mitral E, mitral average e’, and mitral average E/ E’ were assessed.

Two-dimensional speckle-tracking analysis was performed at rest, in the immediate post-exercise period, and in the recovery phase according to the common standard from the consensus document of the EACVI/ASE/Industry Task Force [19]. After manual delineation of the LV endocardial boundary, the software automatically drew the epicardial boundary. Then the widths of the interesting regions were adjusted manually to match the boundaries of the myocardium. The software automatically tracked speckle patterns during the cardiac cycle and yielded a strain curve of the 18 segments of LV. Patients with inadequate tracking of more than one segment in at least one apical view were excluded from the study. LV global longitudinal strain (GLS) was calculated by averaging end-systolic strain of all LV myocardial segments.

### Reproducibility

Intraobserver and interobserver variabilities for LV GLS in the immediate post-exercise period were examined in 10 randomly selected patients. The same observer who was blinded to the initial measurements repeated the measurements after more than 4 weeks to assess intraobserver variability. A second independent observer repeated the measurements twice to assess interobserver variability.

### Statistical analysis

Normality plots with tests were performed using the Shapiro–Wilk test. Continuous variables were presented as the mean ± standard deviation (SD) or median (interquartile range) and categorical variables as percentages. Continuous variables were compared using the independent t-test or Mann–Whitney *U* test, where appropriate. To compare the proportion of categorical variables, chi-square or Fisher exact test was used. Comparisons among ≥3 independent groups were assessed using one-way analysis of variance (ANOVA), and comparisons between groups were performed by post-hoc pairwise comparisons (Scheffe’s). Comparisons among ≥3 matching groups were assessed using one-way repeated measures ANOVA, and post-hoc pairwise comparisons (Tukey’s) were used to probe significant differences between groups. Intraobserver and interobserver variabilities were evaluated by Bland-Altman analysis. For all parameters, *P* < 0.05 (two-tailed) was considered statistically significant. All statistical analyses were performed using SPSS 17.0 software package (SPSS version 17, Chicago, IL, USA).

## Results

**Fig. 1:**
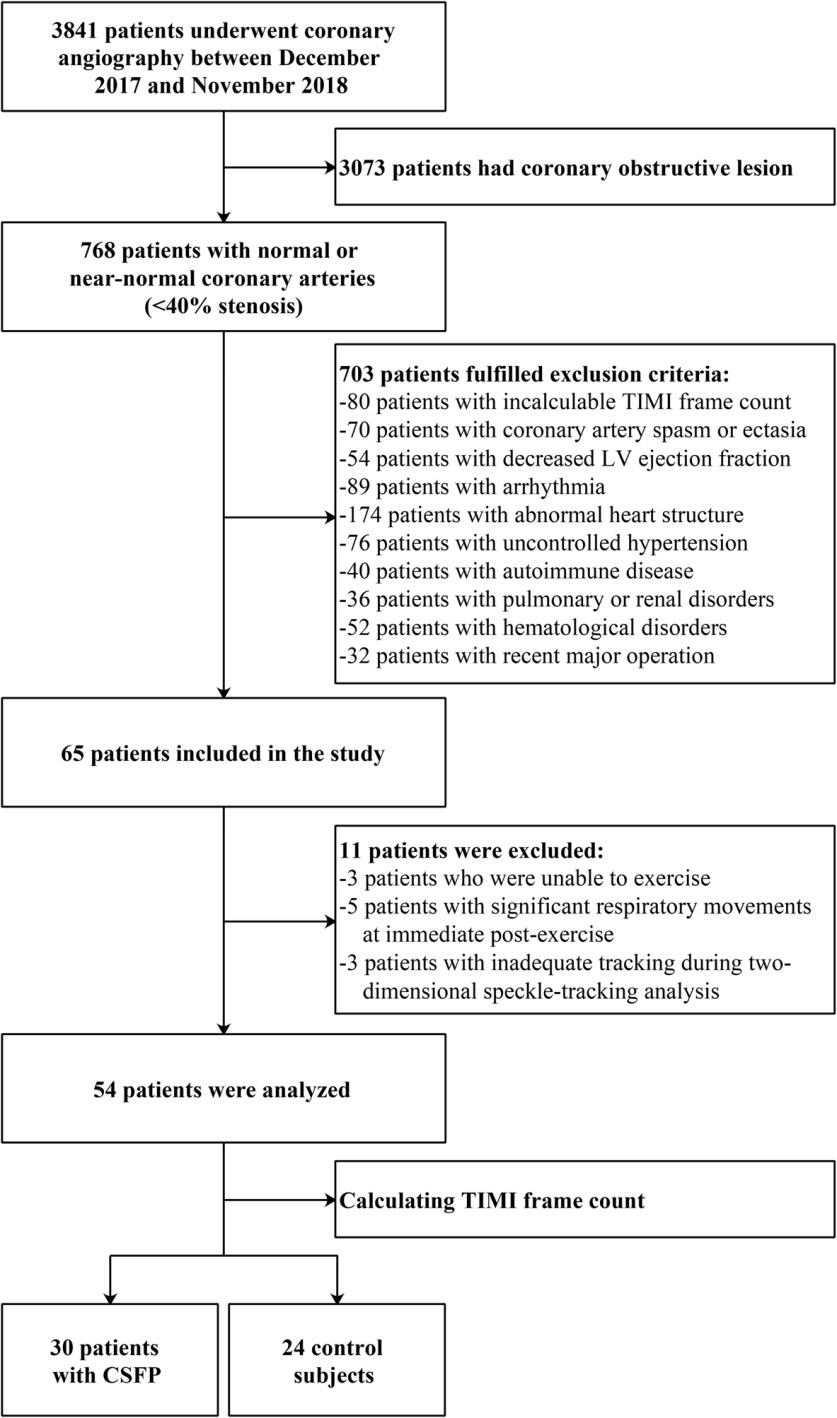
Patient recruitment flow chart. LV, left ventricle

The study flow chart is shown in Fig. 1. A total of 65 patients were enrolled in the study. Of the 65 patients, we excluded 3 patients who were unable to exercise, 5 patients because of significant respiratory movements in the immediate post-exercise period, and 3 patients because of inadequate tracking during two-dimensional speckle-tracking analysis. The analyzed population consisted of 54 subjects (30 patients with CSFP; 24 control subjects).

The demographic, biochemical data, medications, and angiographic findings of the study population are shown in Table 1. All of the individuals did not use intracoronary medications. The red cell distribution width value in patients with CSFP was significantly higher than in controls. Patients with CSFP had significantly higher values of TFC for cLAD, LCx, and RCA as well as mean TFC than controls.

**Table 1:** Comparison of baseline characteristics and angiographic findings

	Controls(*n* = 24)	CSFP(*n* = 30)	OR [95% CI]	*P*-value
Demographics				
Age (yrs)	54.01 ± 8.14	56.31 ± 7.55	1.05 [0.99–1.12]	0.09
Male sex [n(%)]	12 (50%)	20 (67%)	2.00 [0.66–6.03]	0.27
BMI (kg/m^2^)	24.72 ± 3.46	24.13 ± 3.22	0.90 [0.78–1.10]	0.34
Body surface area (m^2^)	1.75 ± 0.15	1.73 ± 0.22	0.92 [0.80–1.12]	0.78
Medical history				
Smoking [n(%)]	5 (21%)	11 (37%)	2.20 [0.64–7.56]	0.24
Systolic blood pressure (mmHg)	127.56 ± 14.26	125.33 ± 11.79	1.01 [0.98–1.05]	0.39
Diastolic blood pressures (mmHg)	76.89 ± 10.06	75.09 ± 8.90	1.02 [0.98–1.07]	0.34
Hypertension [n(%)]	11 (46%)	10 (33%)	0.59 [0.20–1.79]	0.41
Diabetes mellitus [n(%)]	3 (13%)	2 (7%)	0.50 [0.08–3.27]	0.65
Duration of illness (months)	12.0 (5.5–72.0)	16.0 (7.0–48.0)	1.00 [0.99–1.01]	0.63
Laboratory values				
Triglycerides (mmol/L)	1.52 ± 1.00	1.49 ± 0.80	0.96 [0.56–1.67]	0.90
Total cholesterol (mmol/L)	4.43 ± 0.82	4.39 ± 0.76	0.93 [0.49–1.75]	0.82
LDL cholesterol (mmol/L)	2.69 ± 0.69	2.80 ± 0.65	1.28 [0.61–2.70]	0.52
HDL cholesterol (mmol/L)	1.25 ± 0.31	1.13 ± 0.32	0.26 [0.04–1.63]	0.14
Fasting blood glucose (mmol/L)	5.36 ± 0.73	5.54 ± 0.58	1.52 [0.70–3.27]	0.29
Red blood cell count (10^12^/L)	4.54 ± 0.37	4.60 ± 0.44	1.47 [0.41–5.21]	0.56
Red cell distribution width (%)	12.51 ± 0.55	12.91 ± 0.48	6.05 [1.52–24.03]	0.004
Platelet count (10^9^/L)	211.24 ± 38.83	205.15 ± 41.13	0.99 [0.98–1.01]	0.56
Platelet distribution width (%)	12.04 ± 1.70	12.75 ± 1.55	1.31 [0.95–1.81]	0.09
Total bilirubin (μmol/L)	11.87 ± 4.54	10.67 ± 3.51	0.91 [0.82–1.09]	0.12
Uric acid (μmol/L)	292.76 ± 82.76	309.47 ± 64.96	1.01 [0.90–1.08]	0.34
Fibrinogen (g/L)	3.03 ± 0.61	2.88 ± 0.62	0.67 [0.29–1.53]	0.34
Medications				
Aspirin [n(%)]	5 (21%)	4 (13%)	0.58 [0.14–2.47]	0.49
ACEI/ARB [n(%)]	7 (29%)	14 (47%)	2.13 [0.68–6.62]	0.26
β-Blockers [n(%)]	13 (54%)	19 (63%)	1.46 [0.49–4.36]	0.58
Calcium channel blocker [n(%)]	15 (63%)	19 (63%)	1.04 [0.34–3.15]	0.99
Statin [n(%)]	7 (29%)	9 (30%)	1.04 [0.32–3.38]	0.99
Nitrates [n(%)]	15 (63%)	22 (73%)	1.65 [0.52–5.25]	0.56
Levocarnitine/Trimetazidine [n(%)]	12 (50%)	20 (67%)	2.00 [0.66–6.03]	0.27
TFC				
cLAD	23.08 ± 3.55	44.35 ± 15.41	1.41 [1.16–1.73]	< 0.001
LCX	19.90 ± 3.90	33.74 ± 13.86	1.27 [1.11–1.44]	< 0.001
RCA	22.52 ± 3.68	41.13 ± 15.50	1.59 [1.25–2.03]	< 0.001
Mean	21.83 ± 2.54	39.74 ± 12.40	2.03 [1.35–3.06]	< 0.001

Values shown are mean ± *SD* or percentages. Abbreviations: *BMI*, body mass index; *LDL*, low-density lipoprotein; *HDL*, high-density lipoprotein; *ACEI*, angiotensin-converting enzyme inhibitor; *ARB*, angiotensin II receptor blocker; *TFC*, thrombolysis in myocardial infarction frame count; *cLAD*, corrected left anterior descending coronary artery; *LCx*, left circumflex coronary artery; *RCA*, right coronary artery

All patients and controls reached 85% of maximal predicted heart rate. There were no significant arrhythmias, syncope, or deaths during exercise. Heart rate and blood pressure at rest and during exercise did not differ between the CSFP patients and controls (Table 2). Chest pain was experienced by 4 (13%) CSFP patients and by none of the controls. ST-segment depression occurred in 5 (17%) CSFP patients but not in controls. Both chest pain and ST-segment depression occurred in 3 (10%) CSFP patients. In total, positive ExECG was found in 6 (20%) CSFP patients and negative ExECG was found in 24 (80%) CSFP patients. METs were lower in CSFP patients than controls, and CSFP patients with positive ExECG had greater reduction in METs than CSFP patients with negative ExECG (controls, 12.16 ± 1.63; CSFP patients with negative ExECG, 11.44 ± 1.88; CSFP patients with positive ExECG, 9.12 ± 2.04, *P* = 0.01).

**Table 2:** Comparison of exercise stress electrocardiography parameters

	Controls (*n* = 24)	CSFP (*n* = 30)	OR [95% CI]	*P*-value
Peak systolic blood pressure (mmHg)	174.07 ± 23.23	176.54 ± 33.20	1.00 [0.98–1.02]	0.75
Peak diastolic blood pressure (mmHg)	146.86 ± 22.07	148.85 ± 37.07	1.00 [0.98–1.02]	0.81
Peak heart rate (bpm)	150.57 ± 7.70	151.85 ± 9.53	1.02 [0.96–1.08]	0.59
Speed (mph)	4.51 ± 0.51	4.26 ± 0.57	2.39 [0.85–6.72]	0.09
Grade (%)	16.77 ± 1.27	15.86 ± 1.80	1.47 [1.01–2.15]	0.04
Rate–pressure product (10^3^ bpm mmHg)	21.40 ± 3.21	21.33 ± 3.13	1.00 [0.98–1.02]	0.92
METs	12.16 ± 1.63	10.78 ± 2.09	1.52 [1.08–2.13]	0.009
ST-segment depression ≥1 mm [n(%)]	0	5 (17%)		0.04
Angina [n(%)]	0	4 (13%)		0.05
Positive ExECG [n(%)]	0	6 (20%)		0.02

Values shown are mean ± *SD*. Abbreviations: *METs*, metabolic equivalents

**Fig. 2:**
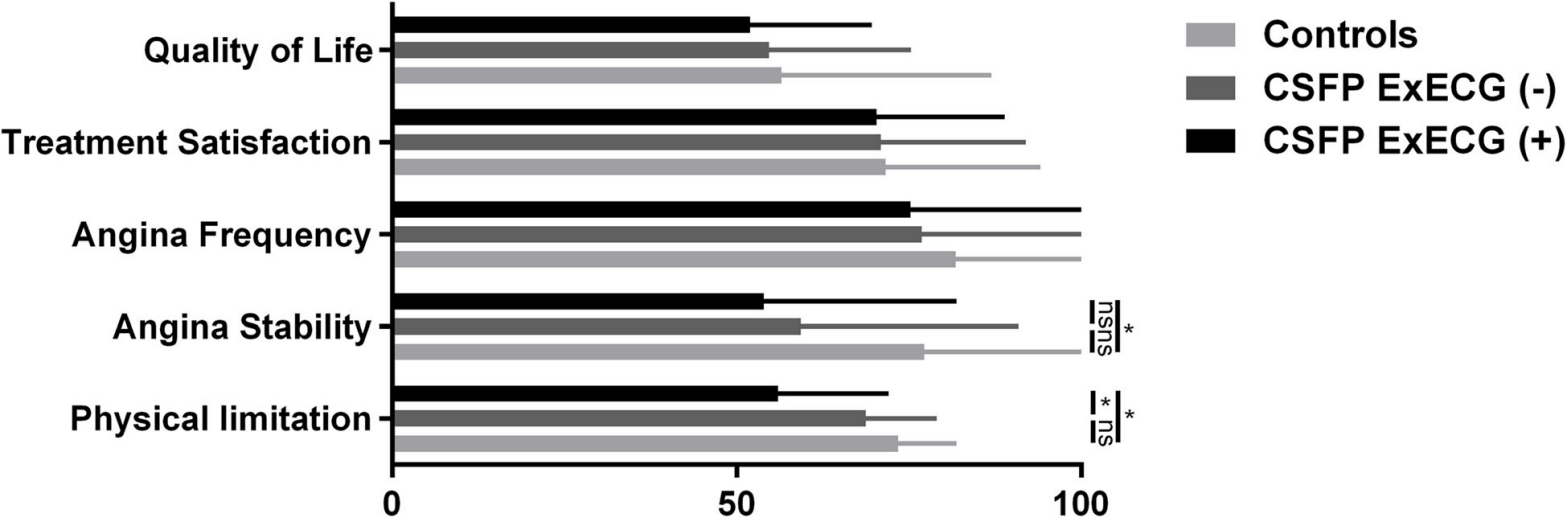
Comparison of Seattle Angina Questionnaire scores on each subscale

Differences in the five subscales of the SAQ among groups are shown in Fig. 2. Patients with CSFP had lower scores on each of the SAQ subscales compared with controls, with significant differences on the SAQ-physical limitation scale and SAQ-angina stability scale. CSFP patients with positive ExECG had greater physical limitations than those CSFP patients with negative ExECG.

Comparison of LV function at rest is shown in Table 3. In the LV systolic function analyses, we observed that the LV GLS in CSFP patients was significantly decreased as compared with controls, but there was no difference between CSFP patients with positive and negative ExECG. Moreover, we found that CSFP patients with positive ExECG had significantly decreased mitral E and mitral average e’ as compared with CSFP patients with negative ExECG and controls, but there were no differences in mitral average E/e’ among groups.

**Table 3:** Comparison of left ventricular systolic and diastolic function at rest

	Controls (*n* = 24)	CSFP ExECG (−) (*n* = 24)	CSFP ExECG (+) (*n* = 6)	*P*-value
LV end-diastolic diameter (mm)	47.12 ± 3.63	47.02 ± 4.87	47.63 ± 2.77	0.83
LV end-systolic diameter (mm)	31.36 ± 3.51	32.39 ± 4.13	32.06 ± 2.80	0.61
LV end-diastolic volume (ml)	80.30 ± 15.02	78.60 ± 13.81	77.52 ± 18.93	0.78
LV ejection fraction (%)	63.00 ± 4.20	63.20 ± 3.70	65.00 ± 4.00	0.15
LV GLS (%)	−21.26 ± 2.74	−19.98 ± 2.32^*^	−19.70 ± 2.38^*^	0.01
LA volume index (ml/m^2^)	27.06 ± 5.12	29.99 ± 3.57^*^	30.56 ± 5.64^*^	0.03
Mitral E (cm/s)	73.85 ± 16.47	72.34 ± 16.65	61.72 ± 15.75^*#^	< 0.001
Mitral A (cm/s)	74.63 ± 16.81	71.28 ± 13.33	67.07 ± 13.59^*^	0.08
Mitral E/A	1.12 ± 0.33	1.09 ± 0.28	0.94 ± 0.21^*^	0.05
Mitral septal e’ (cm/s)	7.40 ± 2.05	6.97 ± 1.76	6.10 ± 1.74^*^	0.01
Mitral lateral e’ (cm/s)	10.41 ± 2.70	9.55 ± 2.18	8.07 ± 2.87^*#^	< 0.001
Mitral average e’ (cm/s)	8.94 ± 2.19	8.26 ± 1.80	7.09 ± 2.14^*#^	< 0.001
Mitral average E/e’	8.59 ± 2.20	8.99 ± 2.09	9.31 ± 2.87	0.33
Tricuspid regurgitation velocity (m/s)	1.66 ± 0.69	1.80 ± 0.54	1.93 ± 0.59	0.29

Values shown are mean ± *SD*. Abbreviations: *LV*, left ventricle; *GLS*, global longitudinal strain; *LA*, left atrium; *E*, early diastolic flow velocity; *A*, late diastolic flow velocity; e’, early diastolic annular velocity

^*^P < 0.05 vs. controls; ^#^*P* < 0.05 vs. CSFP ExECG (−)

**Fig. 3:**
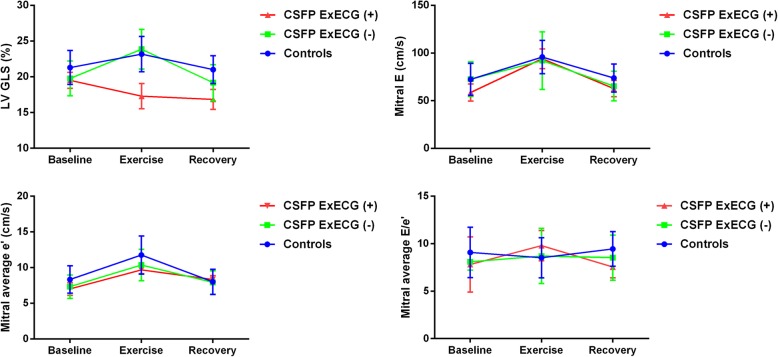
Comparison of left ventricular systolic and diastolic function at rest, during exercise, and recovery phase. GLS, global longitudinal strain; E, early diastolic flow velocity; e’, early diastolic annular velocity

During exercise and recovery phase, no left ventricular wall motion abnormalities were observed in the patients or controls. Mitral E and mitral average e’ increased in all patients and controls during exercise. CSFP patients with negative ExECG and controls had significantly increased LV GLS and decreased mitral average E/e’, but CSFP patients with positive ExECG had significantly decreased LV GLS and increased mitral average E/e’ during exercise (Table 4 and Fig. 3).

**Table 4:** Comparison of left ventricular systolic and diastolic function at rest, during exercise, and recovery phase

	Controls (*n* = 24)	CSFP ExECG (−) (*n* = 24)	CSFP ExECG (+) (*n* = 6)	*P*-value
LV end-diastolic volume (ml)				
Baseline	85.44 ± 17.27	76.23 ± 16.66	76.90 ± 14.58	0.13
Exercise	83.87 ± 17.35	73.96 ± 19.47	77.06 ± 15.67	0.22
Recovery	84.34 ± 16.61	76.42 ± 16.87	77.29 ± 15.54	0.10
*P*-value	0.84	0.82	0.96	
LV ejection fraction (%)				
Baseline	63.67 ± 2.37	63.00 ± 4.00	65.16 ± 3.39	0.11
Exercise	68.00 ± 2.89^*^	68.50 ± 3.29^*^	67.35 ± 2.24	0.37
Recovery	64.00 ± 3.31^#^	63.46 ± 3.88^#^	65.00 ± 2.75	0.30
*P*-value	< 0.001	< 0.001	0.09	
LV GLS (%)				
Baseline	−21.30 ± 2.37	−19.77 ± 2.43	−19.50 ± 1.13	0.05
Exercise	−23.18 ± 2.46^*^	−23.85 ± 2.77^*^	−17.29 ± 1.76^*^	< 0.001
Recovery	−21.00 ± 1.94^#^	−19.17 ± 2.52^#^	−16.84 ± 1.40^*^	< 0.001
*P*-value	< 0.001	< 0.001	0.02	
LA volume index (ml/m^2^)				
Baseline	29.24 ± 7.33	31.08 ± 5.69	31.53 ± 6.56	0.73
Exercise	28.72 ± 7.58	30.48 ± 6.21	31.83 ± 6.11	0.51
Recovery	29.05 ± 7.21	30.91 ± 6.28	31.55 ± 6.00	0.69
*P*-value	0.63	0.70	0.65	
Mitral E (cm/s)				
Baseline	72.42 ± 16.79	72.58 ± 18.10	58.67 ± 8.96	0.17
Exercise	95.83 ± 17.40^*^	92.08 ± 30.27^*^	94.00 ± 10.32^*^	0.86
Recovery	73.83 ± 14.65^#^	65.33 ± 15.52^*#^	62.67 ± 8.50^#^	0.08
*P*-value	< 0.001	< 0.001	< 0.001	
Mitral average e’ (cm/s)				
Baseline	8.33 ± 1.93	7.33 ± 1.63	7.00 ± 0.89	0.08
Exercise	11.75 ± 2.67^*^	10.36 ± 2.19^*^	9.67 ± 0.52^*^	0.06
Recovery	8.00 ± 1.77^#^	7.92 ± 1.64^#^	8.33 ± 0.52^#^	0.85
*P*-value	< 0.001	< 0.001	0.002	
Mitral average E/e’				
Baseline	9.08 ± 2.65	8.09 ± 0.87	7.80 ± 2.90	0.18
Exercise	8.52 ± 2.11	8.70 ± 2.91	9.79 ± 1.60^*^	0.52
Recovery	9.45 ± 1.83	8.53 ± 2.38	7.54 ± 1.14^#^	0.09
*P*-value	0.31	0.37	0.02	

Values shown are mean ± *SD*. Abbreviations: *LV*, left ventricle; *GLS*, global longitudinal strain; *LA*, left atrium; *E*, early diastolic flow velocity; *A*, late diastolic flow velocity; e’, early diastolic annular velocity

^*^P < 0.05 vs. baseline; ^#^*P* < 0.05 vs. exercise

Mean difference in intraobserver variability of LV GLS in the immediate post-exercise period was 0.12% (95% confidence interval [CI]: − 0.92 to 1.13%). Mean difference in interobserver variability was − 0.06% (95% CI: − 1.35 to 1.23%).

## Discussion

In the present study, we evaluated results of ExECG and LV function during exercise by echocardiography in patients with CSFP, and found that (1) about 80% of patients with CSFP exhibited negative ExECG and 20% of patients exhibited positive ExECG; (2) CSFP patients with negative ExECG exhibited improved LV function during exercise but CSFP patients with positive ExECG exhibited impaired LV function; and (3) ExECG can aid in stratification of exercise capacity and LV function in patients with CSFP.

TFC was the only effective and accurate tool for the diagnosis of SCF. Although TFC may be affected by either biologic or technical factors, such as aortic systolic blood pressure, diastolic blood pressures, body surface area, rate of contrast injection or catheter size, we have matche and uniformed the confounders to control the effect on TFC in the study. Currently, the underlying mechanisms of CSFP are not sufficiently clear; they might be related to a chronically elevated resting coronary microvascular resistance resulting from both structural and functional abnormalities [20]. On one hand, endomyocardial biopsies show that there is fibromuscular hyperplasia, myointimal proliferation, mitochondrial abnormalities, endothelial edema, and thickening and degeneration of the coronary microvessels in patients with CSFP [3, 21]. On the other hand, the elevated resting coronary microvascular resistance may be associated with increased red cell distribution width, which is related to impairments in deformability of erythrocytes and changes in blood rheological properties [22].

Our results show that patients with CSFP exhibit differing responses to exercise as depicted by the results of ExECG and LV function measured by echocardiography. The probable explanation may be the different responses of coronary microvascular resistance to exercise in patients with CSFP. Fineschi et al. [23] quantified coronary microvascular resistances using the thermodilution wire at rest and during hyperemia after injecting papaverine in a patient with CSFP with mainly rest angina, and found that the coronary microvascular resistance at rest was elevated but responses to vasodilator stress was normal. Indeed, our study highlighted improved exercise capacity and LV function during exercise stress in the CSFP patients with negative ExECG.

In addition to CSFP, positive ExECG is also observed in cardiac syndrome X, another clinical entity characterized by angina and normal angiograms. However, syndrome X is deemed to have a different presentation and pathophysiology compared with CSFP [24]. CSFP is more common in men presenting with rest angina, but syndrome X commonly affects postmenopausal women with exertional angina. In addition, patients with CSFP mainly have elevated resting resistance but preserved coronary flow reserve (CFR); however, patients with syndrome X have normal resting resistance but reduced CFR [23]. Therefore, although both positive ExECG and normal angiograms are seen in CSFP with positive ExECG and syndrome X, there are essential differences.

ExECG can privode important value for risk stratification in suspected or known coronary heart disease who can exercise, with the advantage of obtaining physiological exercise data, perceived higher feasibility, and superior cost profile compared with other non-invasive tests [11–13]. However, the application of ExECG in patients with CSFP has not been well investigated and its incremental value is not precisely known. Our study found that 80% of patients with CSFP exhibited negative ExECG and 20% of patients exhibited positive ExECG; it further emphasized the incremental value of ExECG for stratification of exercise capacity and LV function. The application of ExECG in patients with CSFP may result in substantial healthcare savings and provide a basis for delivering individualized management to patients.

Unfortunately, there are no standard and effective treatment protocols for patients with CSFP patients, as of now. Recently, several small studies showed that nebivolol can ameliorate symptoms of patients with CSFP through its beta-receptor blocking activity by inducing endothelium-dependent vasodilatation, but its clinical value is limited [25, 26]. Our study revealed that CSFP patients with negative ExECG had significantly improved LV function but CSFP patients with positive ExECG had significantly impaired LV function during exercise. These results may shed light on novel therapeutic modalities in patients with CSFP, such as changing physical activity patterns according to stratification of ExECG. CSFP patients with negative ExECG can be motivated and encouraged to take appropriate exercise, but CSFP patients with positive ExECG should be advised to exercise within reasonable limits. However, larger prospective studies are necessary to verify and validate these results in the future.

### Study limitations

Our study has several limitations. First, the image quality during the exercise stress test limited the absolute feasibility for image analysis in our population. Second, the enrollment of patients from a single center might limit the generalizability of our findings. Third, the lack of invasive CFR might limit further study of the association between CFR and LV function during exercise. Fourth, the sample size of the study population may not have been adequate because of the low prevalence of CSFP, and we are now expanding the sample size and performing a follow-up of these patients.

## Conclusions

About 80% of patients with CSFP exhibited negative ExECG and 20% of patients exhibited positive ExECG. CSFP patients with negative ExECG exhibited improved LV function but CSFP patients with positive ExECG exhibited impaired LV function during exercise. ExECG may aid in the stratification of patients with CSFP according to exercise capacity and LV function. Changing physical activity patterns according to stratification of ExECG may be a novel therapeutic direction in patients with CSFP. This is, however, a preliminary study and larger prospective studies are necessary to verify and validate these results.

## Data Availability

The datasets generated and analysed during the current study are not publicly available due to a further study of this area but are available from the corresponding author on reasonable request.

## Abbreviations

CFR: Coronary flow reserve
CSFP: Coronary slow flow phenomenon
ExECG: Exercise stress electrocardiography
GLS: Global longitudinal strain
LA: Left atrial
LAD: Left anterior descending artery
LCX: Left circumflex coronary artery
LV: Left ventricular
METs: Metabolic equivalents
RCA: Right coronary artery
SAQ: Seattle angina questionnaire
TFC: TIMI frame count
TIMI: Myocardial infarction

## References

[1] Tambe AA, Demany MA, Zimmerman HA, Mascarenhas E (1972). Angina pectoris and slow flow velocity of dye in coronary arteries--a new angiographic finding. Am Heart J.

[2] Singh S, Kothari SS, Bahl VK (2004). Coronary slow flow phenomenon: an angiographic curiosity. Indian Heart J.

[3] Mangieri E, Macchiarelli G, Ciavolella M, Barilla F, Avella A, Martinotti A, Dell'Italia LJ, Scibilia G, Motta P, Campa PP (1996). Slow coronary flow: clinical and histopathological features in patients with otherwise normal epicardial coronary arteries. Catheter Cardiovasc Diagn.

[4] Hawkins BM, Stavrakis S, Rousan TA, Abu-Fadel M, Schechter E (2012). Coronary slow flow--prevalence and clinical correlations. Circ J.

[5] Cutri N, Zeitz C, Kucia AM, Beltrame JF (2011). ST/T wave changes during acute coronary syndrome presentation in patients with the coronary slow flow phenomenon. Int J Cardiol.

[6] Wozakowska-Kaplon B, Niedziela J, Krzyzak P, Stec S (2009). Clinical manifestations of slow coronary flow from acute coronary syndrome to serious arrhythmias. Cardiol J.

[7] Saya S, Hennebry TA, Lozano P, Lazzara R, Schechter E (2008). Coronary slow flow phenomenon and risk for sudden cardiac death due to ventricular arrhythmias: a case report and review of literature. Clin Cardiol.

[8] Wang Y, Ma C, Zhang Y, Guan Z, Liu S, Li Y, Yang J (2015). Assessment of left and right ventricular diastolic and systolic functions using two-dimensional speckle-tracking echocardiography in patients with coronary slow-flow phenomenon. PLoS One.

[9] Wang Y, Ma C, Zhang Y, Guan Z, Liu S, Li Y, Yang J (2016). Layer-specific analysis of left ventricular myocardial contractility in patients with coronary slow-flow phenomenon. J Clin Ultrasound.

[10] Chaudhry MA, Smith M, Hanna EB, Lazzara R (2012). Diverse spectrum of presentation of coronary slow flow phenomenon: a concise review of the literature. Cardiol Res Pract.

[11] Task Force M, Montalescot G, Sechtem U, Achenbach S, Andreotti F, Arden C, Budaj A, Bugiardini R, Crea F, Cuisset T, Di Mario C, Ferreira JR, Gersh BJ, Gitt AK, Hulot JS, Marx N, Opie LH, Pfisterer M, Prescott E, Ruschitzka F, Sabate M, Senior R, Taggart DP, van der Wall EE, Vrints CJ, Guidelines ESCCfP, Zamorano JL, Achenbach S, Baumgartner H, Bax JJ, Bueno H, Dean V, Deaton C, Erol C, Fagard R, Ferrari R, Hasdai D, Hoes AW, Kirchhof P, Knuuti J, Kolh P, Lancellotti P, Linhart A, Nihoyannopoulos P, Piepoli MF, Ponikowski P, Sirnes PA, Tamargo JL, Tendera M, Torbicki A, Wijns W, Windecker S, Document R, Knuuti J, Valgimigli M, Bueno H, Claeys MJ, Donner-Banzhoff N, Erol C, Frank H, Funck-Brentano C, Gaemperli O, Gonzalez-Juanatey JR, Hamilos M, Hasdai D, Husted S, James SK, Kervinen K, Kolh P, Kristensen SD, Lancellotti P, Maggioni AP, Piepoli MF, Pries AR, Romeo F, Ryden L, Simoons ML, Sirnes PA, Steg PG, Timmis A, Wijns W, Windecker S, Yildirir A, Zamorano JL. 2013 ESC guidelines on the management of stable coronary artery disease: the Task Force on the management of stable coronary artery disease of the European Society of Cardiology. Eur Heart J. 2013;34:2949–3003. 10.1093/eurheartj/eht296 23996286

[12] Fihn SD, Blankenship JC, Alexander KP, Bittl JA, Byrne JG, Fletcher BJ, Fonarow GC, Lange RA, Levine GN, Maddox TM, Naidu SS, Ohman EM, Smith PK (2014). 2014 ACC/AHA/AATS/PCNA/SCAI/STS focused update of the guideline for the diagnosis and management of patients with stable ischemic heart disease: a report of the American College of Cardiology/American Heart Association Task Force on Practice Guidelines, and the American Association for Thoracic Surgery, Preventive Cardiovascular Nurses Association, Society for Cardiovascular Angiography and Interventions, and Society of Thoracic Surgeons. J Am Coll Cardiol.

[13] Bourque JM, Beller GA (2015). Value of exercise ECG for risk stratification in suspected or known CAD in the era of advanced imaging technologies. JACC Cardiovasc Imaging.

[14] Gibson CM, Cannon CP, Daley WL, Dodge JT, Alexander B, Marble SJ, McCabe CH, Raymond L, Fortin T, Poole WK, Braunwald E (1996). TIMI frame count: a quantitative method of assessing coronary artery flow. Circulation.

[15] Lang RM, Badano LP, Mor-Avi V, Afilalo J, Armstrong A, Ernande L, Flachskampf FA, Foster E, Goldstein SA, Kuznetsova T, Lancellotti P, Muraru D, Picard MH, Rietzschel ER, Rudski L, Spencer KT, Tsang W, Voigt JU (2015). Recommendations for cardiac chamber quantification by echocardiography in adults: an update from the American Society of Echocardiography and the European Association of Cardiovascular Imaging. J Am Soc Echocardiogr.

[16] Spertus JA, Winder JA, Dewhurst TA, Deyo RA, Prodzinski J, McDonell M, Fihn SD (1995). Development and evaluation of the Seattle angina questionnaire: a new functional status measure for coronary artery disease. J Am Coll Cardiol.

[17] Chan PS, Jones PG, Arnold SA, Spertus JA (2014). Development and validation of a short version of the Seattle angina questionnaire. Circulation. Cardiovasc Qual Outcomes.

[18] Nagueh SF, Smiseth OA, Appleton CP, Byrd BF, Dokainish H, Edvardsen T, Flachskampf FA, Gillebert TC, Klein AL, Lancellotti P, Marino P, Oh JK, Popescu BA, Waggoner AD (2016). Recommendations for the evaluation of left ventricular diastolic function by echocardiography: an update from the American Society of Echocardiography and the European Association of Cardiovascular Imaging. J Am Soc Echocardiogr.

[19] Voigt JU, Pedrizzetti G, Lysyansky P, Marwick TH, Houle H, Baumann R, Pedri S, Ito Y, Abe Y, Metz S, Song JH, Hamilton J, Sengupta PP, Kolias TJ, d'Hooge J, Aurigemma GP, Thomas JD, Badano LP (2015). Definitions for a common standard for 2D speckle tracking echocardiography: consensus document of the EACVI/ASE/industry Task Force to standardize deformation imaging. Eur Heart J Cardiovasc Imaging.

[20] Wang X, Nie SP (2011). The coronary slow flow phenomenon: characteristics, mechanisms and implications. Cardiovasc Diagn Ther.

[21] Mosseri M, Yarom R, Gotsman MS, Hasin Y (1986). Histologic evidence for small-vessel coronary artery disease in patients with angina pectoris and patent large coronary arteries. Circulation.

[22] Akpinar I, Sayin MR, Gursoy YC, Aktop Z, Karabag T, Kucuk E, Sen N, Aydin M, Kiran S, Buyukuysal MC, Haznedaroglu IC (2014). Plateletcrit and red cell distribution width are independent predictors of the slow coronary flow phenomenon. J Cardiol.

[23] Fineschi M, Gori T (2009). Coronary slow flow: description of a new "cardiac Y" syndrome. Int J Cardiol.

[24] Agrawal S, Mehta PK, Bairey Merz CN (2014). Cardiac Syndrome X: update 2014. Cardiol Clin.

[25] Gunes Y, Tuncer M, Guntekin U, Ceylan Y, Sahin M, Simsek H (2009). Regional functions of the left ventricle in patients with coronary slow flow and the effects of nebivolol. Ther Adv Cardiovasc Dis.

[26] Albayrak S, Ordu S, Yuksel H, Ozhan H, Yazgan O, Yazici M (2009). Efficacy of nebivolol on flow-mediated dilation in patients with slow coronary flow. Int Heart J.

[27] Wang Y, Li J, Liu S, Mu L, Li G, Zhao C, Yu H, Yang J, Ma C (2019). 2019 ASE 30th annual scientific sessions-value of treadmill exercise echocardiography combined with stress electrocardiography for Stratifcation of left ventricular systolic and diastolic function in patients with coronary slow flow. J Am Soc Echocardiogr.

